# BiClamp® forcep liver transection versus clamp crushing technique for liver resection: study protocol for a randomized controlled trial

**DOI:** 10.1186/s13063-015-0722-1

**Published:** 2015-04-30

**Authors:** Jiang-ming Chen, Wei Geng, Fu-bao Liu, Hong-chuan Zhao, Sheng-xue Xie, Hui Hou, Yi-jun Zhao, Guo-bin Wang, Xiao-ping Geng

**Affiliations:** Department of Surgery, The Second Affiliated Hospital of Anhui Medical University, No. 678 Furong Road, Shushan District, , Anhui Hefei, 230022 China; Department of Liver Surgery, Ren Ji Hospital, School of Medicine, Shanghai Jiao Tong University, No. 1630 Dongfang Road, Shanghai, 200127 China; Department of Surgery, The First Affiliated Hospital of Anhui Medical University, No. 218 Jixi Road, Shushan District, , Anhui Hefei, 230022 China

**Keywords:** Clamp crushing, BiClamp® forcep transection, Blood loss, Randomized controlled trial

## Abstract

**Background:**

Blood loss and the requirement of blood transfusions during liver transection have been shown to correlate well with higher morbidity and mortality rates and a worse prognosis. Various devices for liver parenchymal transection have been developed to reduce intraoperative blood loss. The goal of this study is to evaluate the safety and effectiveness of BiClamp® forcep transection compared to a clamp crushing technique in patients undergoing liver resection.

**Methods/Design:**

This study will include patients 18 years and older scheduled for hepatectomy with hepatic vascular exclusion who give informed consent. A sample size of 48 patients in each randomization arm will be calculated to detect a difference in the reduction of blood loss of approximately 200 ml (90% power and α = 0.05 (two-tailed)). The primary efficacy endpoint of the trial will be the total intraoperative blood loss based on the randomized dissection technique. The statistical analysis is based on the intention-to-treat population. Patients will be followed up on for three months for complications and adverse events.

**Discussion:**

This prospective, single-center, randomized controlled, single-blinded, two-group parallel trial is designed to assess the efficacy and safety of BiClamp forcep hepatectomy versus clamp crushing for parenchymal transection during elective hepatic resection.

**Trial registration:**

This trial was registered with Clinicaltrials.gov (identifier: NCT02197481) on 15 July 2014.

## Background

Minimization of blood loss during liver resection remains a major concern for hepatic surgeons due to the correlations among excessive hemorrhage, requirement of blood transfusions, higher morbidity and mortality rates and poor long-term outcomes [1-3]. In the last few decades, various techniques have been adopted to decrease intraoperative blood loss during hepatectomy, including inflow occlusion and low central venous pressures. Assuming that inflow occlusion and low central venous pressure (CVP) anesthesia cause significant damage via ischemia and reperfusion [4,5], the method applied for hepatectomy has been considered to be the most critical factor influencing intraoperative blood loss [6].

Since the clamp crushing technique was introduced in the 1980s [7], numerous high-tech equipment has been developed to improve parenchymal transection [8-10]. Randomized clinical trials and a recent meta-analysis have shown that these modern devices result in similar blood loss and transfusion requirements compared to the clamp crushing technique, but with a higher cost due cost of the device and disposable medical apparatus [6,8-10]. The optimal method for liver parenchymal division to obtain minimal blood loss is controversial. Therefore, hepatic surgeons still select the method for liver transection according to their own preferences.

Bipolar vessel sealing (BiClamp® forceps, ERBE Elektromedizin, Tübingen, Germany) is a novel hemostatic device that can seal large tissue bundles and blood vessels up to 7 mm in diameter. The BiClamp forceps resist repetitive autoclaving sterilization, which reduces health care costs and medical waste. In a randomized clinical trial, the application of BiClamp forceps significantly decreased operation time, blood loss and costs in patients undergoing mastectomy and vaginal hysterectomy [11,12]. Our retrospective analysis has provided primary evidence for BiClamp forcep efficacy and safety for liver transection [13]. However, the retrospective non-randomized design of this study could not draw a definitive conclusion. Therefore, we conduct this randomized controlled trial to verify the superiority of the BiClamp forceps during liver transection.

## Methods/Design

### Study aim

The objective of this trial is to compare two different liver transection techniques for liver resection in regards to the total intraoperative blood loss, operation time, liver transection time, intraoperative blood transfusion requirement, reoperation, hepatic injury, duration of postoperative hospital stay, total hospitalization expenditures, mortality and morbidity.

### Patient involvement

**Figure 1:**
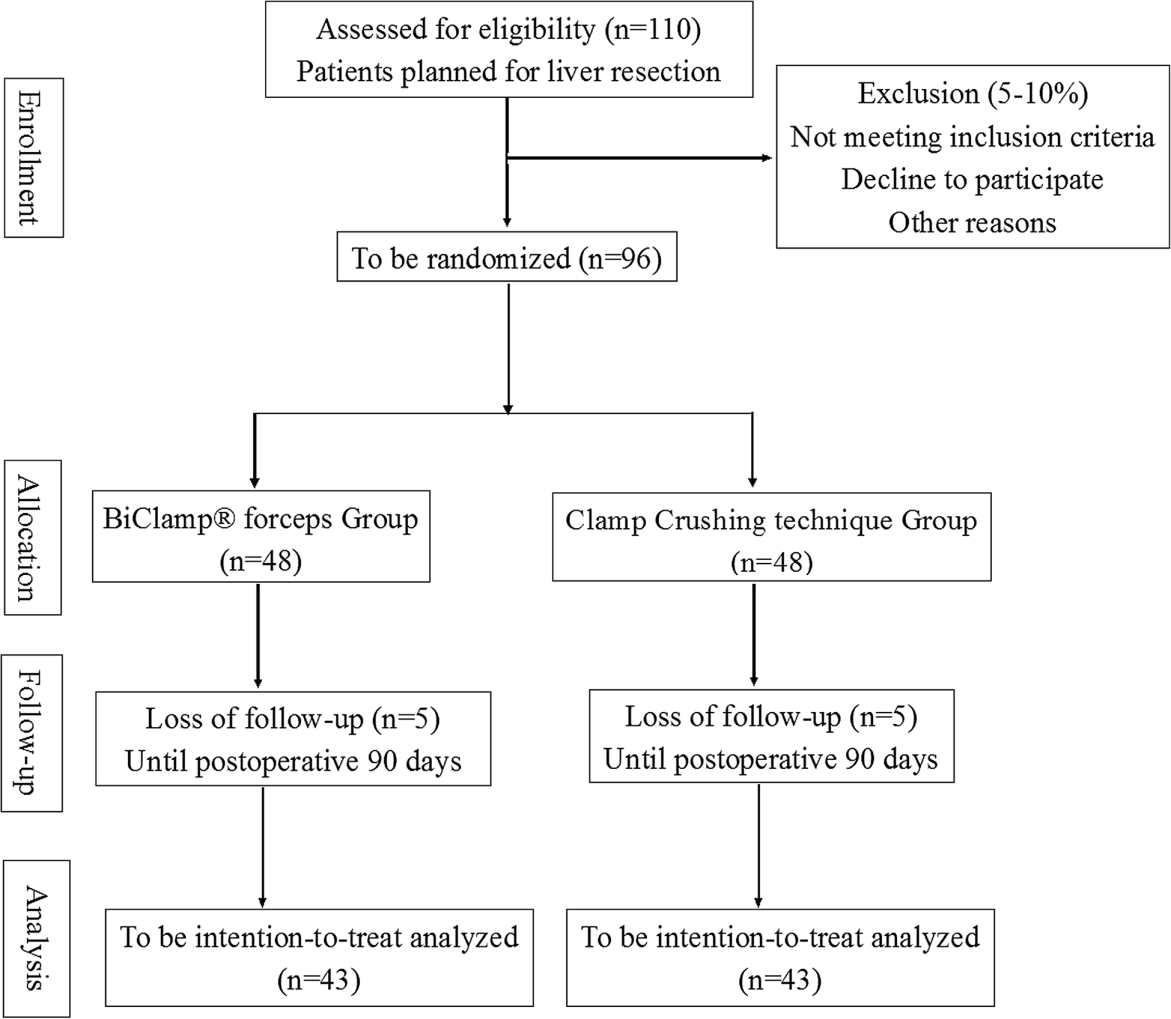
Flowchart according to CONSORT (Figure 1).

The sample size is based on a two-sided t-test for differences with respect to the primary parameter and primary analysis. In a retrospective analysis of our own series, the mean intraoperative blood loss was 450 ml those who underwent BiClamp forceps liver resection, with a standard deviation of 350 ml [14,15]. With a two-sided level of significance α = 5% and a power of 1-β = 90%, a sample size of 43 patients in each group is required to detect a difference in the reduction of blood loss of approximately 200 ml (NCSS and PASS 11 (NCSS, Utah, USA). Assuming an expected withdrawal rate of 10% during the trial, 10 additional patients will be randomized, and a total number of 96 patients need to be enrolled (Figure 1).

### Eligibility criteria

#### Inclusion criteria

Patients who meet the following criteria will be included in the study:

1. Above 18 years of age,
2. Elective hepatic resection due to benign or malignant hepatobiliary disease,
3. Child-Pugh class A or B liver function and
4. Informed consent.

#### Exclusion criteria

Patients who meet any of the following criteria will be excluded from the study:

1. Participation in concurrent intervention trials with interference in the outcome of this study,
2. Laparoscopic hepatectomy,
3. Preoperative liver function evaluation: Child-Pugh class C,
4. Lack of compliance or
5. Pregnancy or lactation.

#### Withdrawal

Patients can withdraw at their own request or at the legal representative’s request at any time during the trial. Patients may also be withdrawn if, in the investigator’s opinion, continuation of the trial may be detrimental to the patient’s wellbeing. The reason for all withdrawals will be recorded in the clinical report forms (CRFs) and in the patient’s medical records. All data will be analyzed according to the intention-to-treat (ITT) principle [16].

### Ethics, study registration and consent

The final protocol was approved by the Ethics Committee of the Second Affiliated Hospital of Anhui Medical University (approval number: KY201406). The trial protocol was registered with the Clinicaltrials.gov protocol registration system (identifier: NCT02197481). All patients who are scheduled for liver resection at the Department of General Surgery, The Second Affiliated Hospital of Anhui Medical University and The First Affiliated Hospital of Anhui Medical University will be screened for eligibility and written informed consent. The study procedure, benefits, risks and data management will be clarified in detail during the preoperative conversation.

### Trial interventions

#### Group A: clamp crushing technique

In the clamp crushing technique group, the parenchymal transection is crushed using Kelly clamps (Geister, Badenia-Wirtembergia, Germany), progressive hemostasis of the vessels with titanium clips (Johnson&Johnson, Chihuahua, Mexico) or ligations, and coagulation by argon beam or electrocauterization **(** ERBE Elektromedizin, Tübingen, Germany**)**. Small vessels (≤1 mm) and minor oozing are controlled with bipolar electrocautery **(** ERBE Elektromedizin, Tübingen, Germany**)**. All other structures, including major intrahepatic bile ducts, are ligated or sutured.

#### Group B: BiClamp forceps hepatectomy

**Figure 2:**
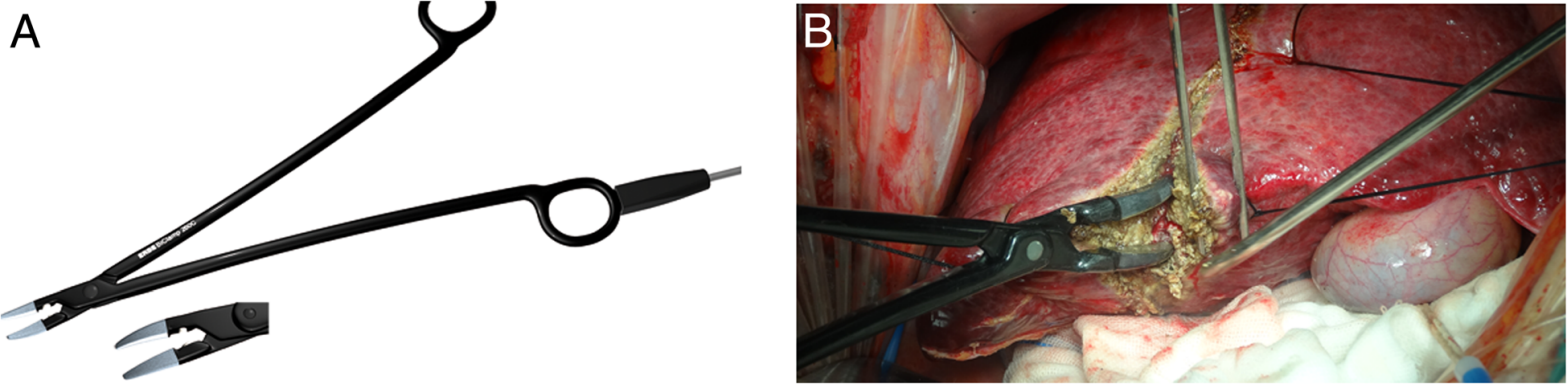
BiClamp forceps liver transection. **A** BiClamp forceps; **B** The surgical process of hepatectomy by BiClamp forceps.

The power of the BiClamp forceps (Figure 2) is set to level four. The underlying liver tissue is divided, and the vessels ≤2 mm in diameter are sealed using the BiClamp forceps. The Glissonian sheaths or hepatic veins with a diameter larger than 3 mm are ligated and divided in the conventional manner to reduce postoperative bile leakage and intraoperative blood loss.

### Primary and secondary endpoints

#### Primary endpoint

The primary efficacy endpoint of the trial will be the total intraoperative blood loss using the randomized dissection technique, which is defined as blood loss from the beginning of the surgical procedure (skin incision) to the end of the surgical procedure (closure of the skin). The amount of blood loss is estimated by including the amount of blood collected in the suction containers, weighing the soaked swabs after subtracting the rinse fluids and ascites, and weighing the dry swabs that were used during operation (assuming that 1 ml of blood is equal to 1 g).

#### Secondary endpoints

Secondary outcomes will be operation time; liver transection time; transection speed; total blood loss per unit transection area; incidence and amount of intraoperative blood transfusion, reoperation and hepatic injury; duration of postoperative hospital stay; mortality; total hospitalization expenditure; and general (wound infection and pulmonary infection) and liver resection associated postoperative morbidity (biliary leakage, post-hepatectomy liver failure and post-hepatectomy hemorrhage) will be assessed (Table 1).

**Table 1:** **Definition of secondary endpoints**

**Secondary endpoint**	**Definition and assessment of outcomes**
Operation time	Time from incision to end of skin closure (minutes)
Liver transection time	Time from beginning to end of liver resection (minutes)
Liver transection area	Area estimated using Adobe Photoshop 7.0 (Adobe System Inc., San Jose, CA, USA) computer software, from the shape of the transection plane, which was traced onto a paper sheet at the end of liver resection (cm^2^)
Liver transection speed	Speed calculated as the transection area divided by the transection time (cm^2^/min)
Total blood loss per unit transection area	Blood loss calculated as the total blood loss divided by the transection area (ml/ cm^2^)
Intraoperative blood transfusion	The indications for blood transfusion were massive hemorrhage (>1,500 ml) during surgery or a hemoglobin level <7 g/dl within stable hemodynamic measures.
Postoperative hospital stay	Time from day of operation until discharge (days)
Total hospitalization expenditure	Costs from admission to discharge ($)
Postoperative liver injury	Serum levels of the aspartate aminotransferase, alanine aminotransferase, bilirubin, albumin and international normalized ratio on postoperative days one, three, five and seven
Mortality	Death due to any cause until 90 days after the operation and the reason
Morbidity	Postoperative complications will be recorded until 90 days after operation. The severity of complications will be graded according to the Clavien-Dindo classification [17].
	Biliary leakage: the international study group of liver surgery (ISGLS) definition (grade A, B or C) [18]
	Post-hepatectomy liver failure: ISGPS definition (grade A, B or C) [19]
	Post-hepatectomy hemorrhage: ISGPS definition (grade A, B or C) [20]
	Intra-abdominal fluid collection or abscess: any imaging modality detecting an intra-abdominal fluid collection associated with abdominal discomfort or pain, or elevation of infectious parameters.
	Pneumonia: infection of the lung with evidence of increased infection parameters (C-reactive protein >2 mg/dl and/or leukocytes >100,000/ml) not caused by a different pathologic process or evidence of pulmonary infiltration on a chest X-ray, requiring antibiotic therapy.

### Type of trial

This is a prospective, randomized, interventional, patient-blinded single center trial with two parallel comparison groups.

#### Randomization

To achieve comparable groups in terms of known and unknown risk factors, randomization will be performed. The allocation schedule will be generated by computer-generated random numbers, with an allocation ratio of 1:1 equal probability of assignment to each group. All patients will be randomized using consecutively numbered opaque envelopes sealed by the investigators. Envelopes will be opened upon entrance of the patient to the operating room.

#### Blinding

Patients and outcome assessors will be blinded to the trial intervention. Blinding of the surgeons and people in the operation is not feasible due to the nature of the interventions.

### Standardization of perioperative care

The operations will be performed by one senior surgeon (XP Geng) who is equally skilled in both the BiClamp forceps and clamp crushing techniques. Except for the trial interventions, perioperative care will be standardized in both study groups following the guidelines of the Department of General Surgery at the Second Affiliated Hospital of Anhui Medical University. In addition, all resections will be performed with the requirement of a low CVP (0 to 5 mm Hg) and an intermittent Pringle maneuver (periods of 15 minutes of clamping and 5 minutes of unclamping).

### Data management and quality assurance

An independent study doctor (JM Chen), who will not be involved in the treatment and monitoring of the patients within the operating room, will enter all required data in the prepared CRF. The CRF should be completed as soon as possible, preferably at the day of the patient’s treatment and visit (Table 2). Reasonable explanations should be given for all missing data. Complete CRF pages will be checked by the principal investigator and the responsible monitor with respect to the completeness and plausibility.

**Table 2:** **Flowchart of the trial**

	**Screening**					
	**Visit 1 Before surgery**	**Visit 2 Day of surgery**	**Visit 3 (POD1)**	**Visit 3 (POD3)**	**Visit 3 (POD7)**	**Visit 4 (POD90)**
Selection criteria and informed consent	×					
Medical history demographics	×					
Physical examination	×					
Laboratory tests	×		×	×	×	×
Trial intervention		×				
Intraoperative outcomes		×				
Postoperative outcomes			×	×	×	×

POD: postoperative days.

### Statistical analysis

The two-sided null hypothesis for the primary outcome measure states that both study interventions lead to similar total intraoperative blood loss; the alternative hypothesis is that one intervention performs better than the other. This null hypothesis will be tested by application of an analysis of covariance that adjusts for the transection surface area, CVP, preoperative international normalized ratio (INR) and preoperative platelet count.

Background characteristics and surgical outcome measures will be compared using Fisher’s exact tests or chi-squared for categorical data, and Student’s t-test for continuous variables. Categorical data will be presented as frequencies and group percentages, and continuous variables will be expressed as the means and standard deviations. The homogeneity of the two groups will be described by comparing the demographic data and baseline values. All analyses will be performed on an ITT basis [16]. For the ITT analysis, data will be processed for all trial patients in the groups in which they are randomized. Statistical significance is defined as *P* <0.05. All statistical calculations will be performed with the help of SPSS10.0 (SPSS, Chicago, USA).

## Discussion

Since the introduction of the clamp crushing technique in 1973 [7], this technique has become the gold standard for liver parenchymal transection due to its effectiveness in controlling blood loss, reduction in operative time and low cost. A variety of devices have been developed to improve parenchymal transection in the past 10 to 15 years [8-10]. However, many randomized studies have failed to show any significant advantage over the clamp crushing technique [8-10]. BiClamp forceps, a reusable bipolar sealing instrument, has been available for open surgery procedures since 2002 [11]. Our retrospective data from between July 2007 and July 2014 has shown a significant reduction in blood loss and lower costs in patients operated on using BiClamp forceps. Based on these results, the first randomized controlled trial is designed to evaluate the efficacy, safety and cost-effectiveness of BiClamp forceps compared to the clamp crushing technique during elective hepatic resection.

## Trial status

The study protocol has been completed in July 2014. Enrollment started on 1 October 2014. XP Geng performs approximately 80 hepatectomies per year. The estimated time frame to randomize 48 patients will be approximately 20 months.

## Abbreviations

CRFs: Clinical Report Forms
CVP: Central venous pressure
INR: International normalized ratio
ITT: Intention-to-treat
